# 30 Days Wild and the Relationships Between Engagement With Nature’s Beauty, Nature Connectedness and Well-Being

**DOI:** 10.3389/fpsyg.2018.01500

**Published:** 2018-09-03

**Authors:** Miles Richardson, Kirsten McEwan

**Affiliations:** Human Sciences Research Centre, University of Derby, Derby, United Kingdom

**Keywords:** nature, nature connectedness, emotion regulation, beauty, restoration, well-being

## Abstract

Recent research suggests that engagement with natural beauty (EWNB) is key to the well-being benefits of nature connectedness. The Wildlife Trust’s *30 Days Wild* campaign provides a large-scale intervention for improving public engagement with nature and its beauty. The effect of *30 Days Wild* participation on levels of EWNB and the relationship between EWNB, nature connectedness and happiness was evaluated during the 2017 campaign. Of the 49,000 people who signed up to the campaign, 308 people fully completed measures of EWNB, nature connection, health, happiness, and conservation behaviors at baseline, post-30 days and post-2 months. There were sustained and significant increases for scores in nature connection, health, happiness, and conservation behaviors. In addition, *30 Days Wild* was the first intervention found to increase EWNB. Further, the significant increase in EWNB mediated the relationship between the increases in nature connectedness and happiness. In a supplementary study to understand the well-being benefits further (*n* = 153), emotional regulation was found to mediate the relationship between nature connectedness and happiness, but EWNB and emotional regulation were not related. The links between nature’s beauty, nature connectedness and well-being are discussed within an account of affect-regulation.

## Introduction

*“The exceeding beauty of the earth, in her splendour of life, yields a new thought with every petal. The hours when the mind is absorbed by beauty are the only hours when we really live"* *Richard Jefferies, “The Pageant of Summer."*

The beauty of nature is a fundamental aspect of the human relationship with the wider natural world. Our cultural history contains continual references to nature’s beauty, and aesthetics have long been considered by research into human–nature relationships. Kaplan (1987) proposed that human preference for natural scenes has an evolutionary basis; our attentional resources were attuned to cues within the natural environment in order to enhance our survival. Therefore, humans have a preference for natural forms and Ulrich (1983) argued that our aesthetic response to natural forms is central to our understanding of human–nature relationships. Kellert’s nine values of Biophilia also include an aesthetic dimension (Kellert, 1993). More recently, engagement with natural beauty (EWNB) has been noted as a key factor in the well-being benefits nature brings (Zhang et al., 2014a). This paper will briefly consider beauty, before introducing nature’s beauty and the relationship with nature connectedness and well-being within the context of affect regulation. Results from an evaluation of a large-scale public engagement with a nature campaign, “*30 Days Wild,”* will be presented considering the effect of taking part in *30 Days Wild* on happiness, health, conservation behaviors, nature connectedness, and EWNB. To further the understanding of well-being benefits of nature connectedness the paper also considers the relationship between happiness, EWNB, nature connectedness, and emotional regulation.

### Beauty and Its Benefits

Beauty has been a topic of human thought for millennia, with Western philosophy considering beauty as a fundamental aspect of human being. Beauty is a perceptual experience of fluency and resulting pleasure and it has been suggested that the same psychological processes underlie judgements of beauty and truth (Reber et al., 2004). Beauty provides pleasure without utility and before reasoning, yet Kaplan (1987) noted how aesthetics guide human behavior with far-reaching consequences. Beauty lies within the characteristics of the object, and the interaction between the object and the person’s cognitive and affective processes. Diessner and Steiner (2017) note that although love and beauty are inextricably linked, the importance of beauty has to be defended.

Research evidence shows that an appreciation of beauty generally (rather than specifically natural beauty) is positively associated with prosociality and well-being (Martínez-Martí et al., 2016). In an online empirical study, Proyer et al. (2016) found increased levels of happiness at three time points after participants noted “beautiful things” in human behavior, nature and generally, the design did not allow the functional type of beauty to be identified. Given the benefits, there have been attempts to develop interventions to improve the appreciation of beauty, although Proyer et al. (2016) noted a lack of intervention studies on appreciation of beauty, both human and nature focussed. Martínez-Martí et al. (2014), using a qualitative evaluation, found that a 3-week web-based intervention improved well-being and appreciation of beauty generally.

### Nature’s Beauty

Rather than nature’s beauty, the focus of Western philosophy has tended to focus on beauty in art (Diessner et al., 2008). As noted above, an evolutionary basis is theorized to account for the human preference for natural scenes. In the first published study focussing on improving EWNB, Diessner et al. (2015) found that ten “directed-attention beauty walks” increased the noticing of natural beauty, but no significant difference in trait EWNB was found. Diessner and Steiner, 2017 found that an intervention could increase overall appreciation of beauty, but once again this did not produce a significant increase in EWNB. These studies used the Engagement with Beauty scale developed by Diessner et al. (2008). This scale includes an EWNB sub-scale with questions on noticing nature’s beauty, but also emotional and spiritual feelings, and the physical feelings when perceiving beauty in nature that can be related to pre-cognitive physiological responses and affect. Diessner suggests that the scale measures trait engagement with beauty, and such traits by definition are stable across time and environments.

### Nature’s Beauty and Nature Connectedness

A small body of recent research has indicated that EWNB is key to the well-being benefits of nature connectedness, a psychological construct that describes a closer affective relationship with nature. Indeed, Zhang et al. (2014a) stated that “connectedness with nature only predicts well-being when individuals are also *emotionally* attuned to nature’s beauty” (p. 55). However, although aesthetics is included as a value of biophilia (Kellert, 1993), there is limited understanding of the links between nature connectedness and natural beauty. Gregory Bateson proposed that greater connection to nature and the wider ecology depended upon aesthetic experience (Charlton, 2008). In a thematic analysis of a personal journey, Richardson and Hallam (2013) found that nature connectedness reflected a personal fulfillment in the landscape that was manifested through an engagement with the beauty of nature. It has also been found that nature’s beauty is often seen as a “good thing” in everyday nature (Richardson et al., 2015). Lumber et al. (2017) found that engagement with aesthetics within nature consistently mediated the relationship between the moralistic values and nature connectedness.

Returning to the role of nature’s beauty and nature connectedness in well-being, Zhang et al. (2014a) found that the positive relationship between a connection with nature and satisfaction with life was only significant for those people attuned and engaged with nature’s beauty. People who experience positive emotion when seeing beauty in nature have higher well-being. Secondly, Zhang et al. (2014b) found that pro-social, or helping behaviors such as empathy and generosity were, once again, found to be linked to engagement with nature’s beauty. First, in those people disposed to perceive beauty in nature, and then to people exposed to beautiful images of nature. More recently, Capaldi et al. (2017) investigated the relationship between nature connectedness and EWNB in three cultures, Canadian, Japanese, and Russian students. They found that EWNB and nature connectedness were positively associated with well-being measures. Their analysis suggested that EWNB has a positive affect on well-being through promotion of a stronger connection with nature. They also noted more support for a mediation model, rather than Zhang’s moderation account.

### Nature’s Beauty, Connectedness, Affect-Regulation, and Well-Being

There is a need to understand how being emotionally attuned to nature’s beauty and nature connectedness are related to well-being. A body of emotional regulation research evidences the links to well-being (Gross, 2013; DeSteno et al., 2013). Korpela et al. (2018) note how the role of nature in affect regulation is often overlooked and describe the relationship between affect regulation and well-being. Korpela et al. (2018) call for further study into environmental affect regulation strategies. Richardson et al. (2016b) demonstrated how responses to nature exposure can be linked to affect regulation by considering the three-circle model of emotion (Gilbert, 2009). Similarly, research into responses to engaging with nature’s beauty by Song et al. (2017) and Chirico et al. (2017) match those observed during forest bathing and accounted for by the three-circle model. The three-circle model contains three dimensions of our affect regulation system that help explain how we can experience threat, drive and contentment.

Ulrich (1983) provides further insight into the relationship between aesthetic and affective response to nature, noting that affect precedes cognition, we feel before we think when sensing nature. The eventual cognitive appraisal of the scene is informed by both the initial affective reaction and by culture and experience to create a post-cognitive affective state which impacts on motivation, action, and behavior.

Previous research suggests that affective response to nature’s beauty will mediate the relationship between nature connectedness and the positive affect based well-being outcome of happiness. Finally, the construct of nature connectedness also has a basis in affect and is associated with a range of well-being benefits (for a review see Richardson et al., 2017). As Capaldi et al. (2017) suggest that nature connectedness is the route by which EWNB brings well-being, there is a need to consider the mechanism by which nature connectedness brings well-being. Given the suggestion, supported by the findings of Gidlow et al. (2016), that the well-being benefits of nature connectedness are not adequately described by attention-restoration (ART) and stress reduction theories developed to explain the benefits of nature exposure (Capaldi et al., 2017) the present paper also considers potential links between nature connectedness, well-being and affect regulation through data from a supplementary study. This also allows the relationship between EWNB and affect regulation to be considered.

### 30 Days Wild

*30 Days Wild* is a large-scale longitudinal nature engagement campaign developed by The Wildlife Trusts to encourage people in the United Kingdom to value nature more highly during their everyday living. It engages people with nature by asking them to interact with nature every day for one month. A wide range of potential activities are suggested across various themes and levels. The four main types are noticing (e.g., take a moment to watch a butterfly), sharing (e.g., sharing experiences and feelings via social media), doing (e.g., pro-nature behaviors such as leaving a wild area in the garden) and connecting (e.g., nature based arts). These vary in resource requirements, level of dedication and time required to provide 101 “Random Acts of Wildness” on a dedicated website and campaign booklet. As a live campaign, The Wildlife Trusts also worked to encourage participation throughout the campaign using specific *30 Days Wild* social-media accounts and blogs. These were very active, with 107,522 #30DaysWild tweets, 29,669 Instagram photos posted and 11,523 Facebook group users. In 2015 12,400 people signed up for *30 Days Wild*, followed by 25,000 people in 2016. The previous published evaluation presented the data from the first year of the campaign (2015 data) and found sustained increases in happiness, health, connection to nature and pro-nature behaviors (Richardson et al., 2016a). The present evaluation focuses on the data from the third year of the campaign (2017) and asks a number of research questions to replicate and extend the previously found effects on happiness, health, connection to nature and pro-nature behaviors by including a measure of EWNB in the standard in-campaign evaluation questions for the first time. Then, to extend the understanding of the campaign benefits in the context of emotion regulation, supplementary data was collected given the restrictions of the in-campaign evaluation. Therefore, the present paper asks three research questions, two within the evaluation: (i) Does taking part in *30 Days Wild* have an effect on happiness, health, conservation behaviors, nature connectedness and EWNB? (ii) Does EWNB mediate the relationship between nature connectedness and well-being? A third research question is addressed in a supplementary study: (iii) What is the relationship between nature connectedness, EWNB, happiness and emotional regulation?

## Materials and Methods

### Design

As detailed in previous *30 Days Wild* work (Richardson et al., 2016a), the evaluation uses a 1 × 3 (A-B-B) repeated measures design with self-reported scores taken at three time-points: pre-participation, post-participation and follow-up at 2 months post completion. The approach has a long history of successful use in non-medical research (Sanson-Fisher et al., 2007), particularly where an intervention has little potential for harm (Bonell et al., 2011). The clear rationale, theoretical basis, and defined outcomes, meet public health intervention checklist criteria (Des Jarlais et al., 2004). The approach is able to provide strong evidence that an intervention is effective within a public health context (Rychetnik et al., 2002). As with similar large-scale health promotion campaigns (Pollard et al., 2008) and applied nature intervention evaluations (Bruni et al., 2017) a randomized controlled trial (RCT) was not a practical option. Further, the chosen design approach is known to be acceptable when measures are relatively stable over time (Bonell et al., 2011). For example, in the United Kingdom, happiness remains constant through the summer with variation in early Spring and late Autumn being small (e.g., approximately 1%; ONS, 2012).

The tone and length of the standard in-campaign evaluation means traditional psychometric scales aren’t suitable for inclusion. Therefore, to supplement the evaluation and answer the third research question, a short cross-sectional supplementary study including a measure of emotional regulation was conducted to explore the links between emotion regulation, nature connectedness and EWNB. This data was collected via an online questionnaire from a separate sample recruited via social-media and Internet discussion forums.

### Participants

Of the 49,000 people who formally signed up for the June 2017 running of *30 Days Wild*, 8,442 (93.7% White, 6.3% other or not stated) aged between 18 and 85 successfully completed the baseline pre-participation survey during the sign-up process (e.g., May 2017). The mean age was 43.37 (*SD* = 12.78), with 1,098 males and 7,344 females. Three-hundred and eight people (93.2% White, 6.8% other or not stated) progressed and responded to invitations to complete both the post-participation survey in July and the follow-up survey in September. The mean age was 49.51 (*SD* = 14.17), with 48 males and 260 females. A further 153 participants aged between 18 and 75 took part in the cross-sectional supplementary study. The mean age was 45.78 (*SD* = 11.74) with 97 females and 56 males taking part.

### Materials

As detailed in previous *30 Days Wild* work (Richardson et al., 2016a), a survey framed as a “Wildness Quiz” was used to evaluate the affect of the campaign on participants. As a public engagement campaign, the communications style was maintained in order to engage participants and this, together with the framing as a “Wildness Quiz,” also had the benefit of helping to reduce potential demand characteristics. The style and purpose of the campaign required that the survey could not be extensive and include traditional psychometric scales. Single item measures are routinely used to monitor population well-being by the United Kingdom’s Office for National Statistics (ONS, 2012).

In addition to questions about age and gender, the survey measured nature connectedness, EWNB, health and well-being and pro-nature behavior. A single question on general happiness, “In general, do you feel happy?” with an 11-point scale response was used to measure well-being. It offers a reliable and valid measure of well-being for community surveys (Abdel-Khalek, 2006) and has been shown to correlate highly with multi-item well-being scales (e.g., Oxford Happiness Index and Satisfaction with Life Scale). Similarly, a single item worded, “In general, would you say your health is” was used to measure health with participants responding on a 5-point rating scale from Poor to Excellent. This approach has been used successfully in previous research, for example, Ostrove et al. (2000). Nature connectedness was measured with an online implementation of the single item inclusion of nature in self (INS) scale (Schultz, 2001). The standard wording was used, with the question introduced as being about “you and nature,” with a short definition of nature provided. The INS represents “self” and “nature” within two circles. Participants select the level of overlap, or interconnection that best describes their relationship and interconnection with the natural environment. The 4-item natural beauty sub-scale from the Engagement with Beauty scale developed by Diessner et al. (2008) was also used with participants responding on a 7-item “very unlike me” to “very much like me” scale. The four questions are: I notice beauty in one or more aspects of nature; When perceiving beauty in nature I feel changes in my body, such as a lump in my throat, an expansion in my chest, faster heart beat, or other bodily responses; When perceiving beauty in nature I feel emotional, it “moves me,” such as feeling a sense of awe, or wonder or excitement or admiration or upliftment; When perceiving beauty in nature I feel something like a spiritual experience, perhaps a sense of oneness, or being united with the universe, or a love of the entire world. Pro-nature conservation behavior was measured via five questions that asked about participants’ actions using “Yes, I do this” and “No, I don’t do this” as response options. The five questions were: I put food out to feed garden birds; I move insects if they are in danger; I grow flowers and plants that birds and insects will like; I am a member of a wildlife or nature organization (e.g., Wildlife Trust, RSPB, WWF, etc.); I do conservation work away from home (e.g., Wildlife Trust Volunteer, etc.).

As noted, the short “Wildness Quiz” format of the in-campaign evaluation means traditional psychometric scales aren’t suitable for including in the main evaluation. Therefore, the difficulties in emotion regulation scale (DERS) was included in a supplementary study. This study adapted the consent and debrief information in the main evaluation and included all of the above measures plus the 16-item DERS (Bjureberg et al., 2016). DERS has been found to be associated with measures relevant to the benefits of nature connectedness and affect regulation (e.g., generalized anxiety disorder and psychophysiological measures such as heart rate variability; Berna et al., 2014).

### Ethics Statement

All participants provided informed consent, recorded via an online tick box labeled “Yes – I accept” that followed a written brief on a “Your Consent” page. The Psychology Research Ethics Committee at the University of Derby approved the evaluation and consent procedure. The Ethics Committee at the University of Derby also approved the supplementary study including the additional measure.

### Procedure

As detailed in previous *30 Days Wild* work (Richardson et al., 2016a), invitations to answer the questions were included in the sign-up process for *30 Days Wild*. Following the consent page participants completed the questions before being provided with a short debrief. Participants then took part in their selected *30 Days Wild* activities during June, in an unmonitored fashion. Participants who had completed the pre-participation survey were invited by e-mail to complete the post-participation and follow-up surveys in July and September. The cross-sectional supplementary study used opportunity sampling with participants following a link to an online consent page and debrief adapted from the main study.

### Data Analysis

SPSS version 24 was used for all analyses. Differences between pre and post participation, and pre and follow-up results were investigated using paired samples *t*-tests. A 1 × 3 (Time) repeated measures ANOVA was used to investigate differences between all three time points. To explore the relationship between changes in nature connectedness, happiness, engagement with nature’s beauty and emotion regulation, mediation analyses were conducted. As it has more power than the sobel or causal steps tests, a bootstrapping approach with 5,000 bootstrap re-samples at a 95% confidence interval was used (Hayes, 2009).

## Results

### Pre-participation Baseline Analysis

Mean and standard deviations by gender at baseline are provided in **Table 1**. Owing to the large disparity in participation between genders *t*-tests were conducted to ascertain if there are any differences that might provide an explanation. Significant differences at *p* < 0.001 are indicated in **Table 1**. Pearson correlations between the main measures were conducted and all were significant (*p* < 0.01; **Table 2**). Correlations repeated by gender are not included as significant results were identical and level of associations similar.

**Table 1:** Mean and standard deviations for baseline measures by gender.

	Female			Male			Total		
	Mean	*N*	*SD*	Mean	*N*	*SD*	Mean	*N*	*SD*
Age	42.85^*^	7,344	12.54	46.86^*^	1,098	13.75	43.37	8,442	12.78
Nature Connection	50.50^*^	7,344	26.49	56.25^*^	1,098	28.31	51.25	8,442	26.8
Conservation Behaviors	2.62^*^	7,344	0.88	2.74^*^	1,098	0.92	2.64	8,442	0.88
Health	3.6	7,344	0.94	3.65	1,098	0.92	3.61	8,442	0.93
Happiness	7.24	7,344	1.7	7.22	1,098	1.78	7.24	8,442	1.71
EWNB	23.39^*^	7,344	3.97	22.33^*^	1,098	4.46	23.25	8,442	4.05

^∗^*Difference between males and female significant at the 0.01 level (2-tailed). EWNB, engagement with natural beauty.*

**Table 2:** Correlation matrix for baseline measures.

	Nature connection	Conservation behaviors	Health	Happiness	EWNB
Nature connection	1				
Conservation behaviors	0.284^∗∗^	1			
Health	0.080^∗∗^	0.079^∗∗^	1		
Happiness	0.157^∗∗^	0.154^∗∗^	0.507^∗∗^	1	
EWNB	0.312^∗∗^	0.270^∗∗^	0.060^∗∗^	0.133^∗∗^	1

^∗∗^*Correlation is significant at the 0.01 level (2-tailed). EWNB, engagement with natural beauty.*

### Does Taking Part in 30 Days Wild Have an Effect?

ANOVA analysis and pairwise comparisons revealed statistically significant increases from pre-participation baseline to post-participation were found for EWNB, nature connectedness, health, happiness, and conservation behaviors (**Tables 3**, **4**). Similarly, there were significant increases from pre-participation baseline to follow-up for the same measures (**Tables 3**, **4**).

**Table 3:** Pre, post-participation and follow-up mean and standard deviations for the four outcome measures.

	Pre-participation		Post-participation		Follow-up	
	Mean	*SD*	Mean	*SD*	Mean	*SD*
Connection to nature	56.75	25.93	64.29	23.99	64.42	22.80
Conservation behaviors	2.82	0.83	2.95	0.73	2.93	0.76
Health	3.62	0.96	3.76	0.93	3.82	0.95
Happiness	7.50	1.58	7.78	1.51	7.87	1.49
EWNB	23.88	3.43	24.38	3.43	24.51	3.38

*EWNB, engagement with natural beauty.*

**Table 4:** Summary of paired *t*-tests and repeated measures ANOVA analyses.

	Pre to Post		Pre to Follow-up		1 × 3 ANOVA		
	*T*(307)	*d*	*T*(307)	*d*	*F*	*df*	η^2^
Connection to nature	5.15	0.30	5.03	0.31	20.54	1.75, 536.00	0.06
Conservation behaviors	4.23	0.17	3.05	0.14	9.70	1.86, 572.12	0.03
Health	3.77	0.15	5.07	0.21	14.73	2, 614	0.05
Happiness	4.03	0.18	5.07	0.24	16.08	1.92, 591.73	0.05
EWNB	3.37	0.15	4.01	0.19	10.22	1.94, 596.17	.03

*All significant at p < 0.01. EWNB, engagement with natural beauty.*

### Does EWNB Mediate the Relationship Between Nature Connectedness and Well-Being?

Following previous research (e.g., Capaldi et al., 2017) mediation analysis on the pre to post-participation change data was conducted using improvement in nature connectedness as a predictor of improvement in happiness, with improvement in EWNB as a mediator. The model met the criteria for mediation (Baron and Kenny, 1986), with both sobel and bootstrap results showing the indirect effect to be significant (**Table 5**).

**Table 5:** Mediation analysis for changes in happiness, nature connection, and EWNB (5000 Bootstrap Samples).

	*β*	*SE*	*t*	*p*
Nature connection to happiness: Total effect	0.009	0.003	3.273	<0.01
Nature connection to EWNB	0.017	0.006	2.914	<0.01
EWNB to happiness controlling for nature connection	0.075	0.026	2.904	<0.01
Nature connection to happiness controlling for EWNB: Direct effect	0.007	0.003	2.791	<0.01

	***Z***	***p***	**LL95%CI**	**UL95%CI**

Indirect sobel and bootstrap effects	2.000	0.046	0.0001	0.003

*EWNB, engagement with natural beauty.*

To provide wider context for the mediation analysis and to explore the relationship between the various measures and improvement in happiness over June, multiple regression analysis was used. The independent variables (IVs) or predictors were all baseline measures and age in the first block, followed by changes in nature connection and EWNB with change in happiness as the DV. The results show that the model including the change in nature connection and EWNB accounted for 23.9% of the variance in happiness improvement, with *R* = 0.51 and Adjusted *R*^2^ = 0.24, *F*(9,298) = 11.703, *p* < 0.01. See **Table 6** for a breakdown of IV results.

**Table 6:** Predictors of improvement in happiness.

	*β*	*SE*	*t*	*p*
Age	0.021	0.005	0.386	0.700
Baseline nature connectedness	-0.046	0.003	-0.653	0.514
Baseline health	0.128	0.072	2.220	0.027
Baseline conservation behaviors	-0.047	0.079	-0.864	0.388
Baseline EWNB	0.180	0.024	2.601	0.010
Baseline happiness	-0.479	0.047	-7.745	0.000
Change in nature connectedness	0.076	0.003	1.204	0.230
Change in EWNB	0.213	0.026	3.706	0.000

*EWNB, engagement with natural beauty.*

### What Is the Relationship Between Nature Connectedness, EWNB, Emotional Regulation, and Happiness?

Pearson correlations were conducted between the measures of nature connectedness (INS), emotional regulation (DERS), EWNB and happiness (single-item) administered in the supplementary study and are shown in **Table 7**. This analysis suggested that the relationship between nature connectedness, happiness, and emotional regulation could be explored further using mediation analysis. Nature connectedness was entered as a predictor of happiness, with emotional regulation as a mediator. The model met the criteria for mediation, with both sobel and bootstrap results showing the indirect effect to be significant (**Table 8**).

**Table 7:** Correlation matrix for the supplementary measures.

	EWNB	Nature connection	DERS	Happiness
EWNB	1			
Nature connection	0.432^∗∗^	1		
DERS	0.004	-0.297^∗∗^	1	
Happiness	0.117	0.318^∗∗^	-0.549^∗∗^	1

^∗∗^*Correlation is significant at the 0.01 level (2-tailed). N = 153.*

*EWNB, engagement with natural beauty; DERS, difficulties in emotion regulation scale.*

**Table 8:** Mediation analysis of nature connection, DERS and happiness (5000 Bootstrap Samples).

	*β*	*SE*	*t*	*p*
Nature connection to happiness: Total effect	0.360	0.088	4.120	<0.01
Nature connection to DERS	-2.317	0.606	-3.821	<0.01
DERS to happiness controlling for nature connection	-0.073	0.010	-7.121	<0.01
Nature connection to happiness controlling for DERS: Direct effect	0.192	0.080	2.422	<0.05

	***Z***	***p***	**LL95%CI**	**UL95%CI**

Indirect sobel and bootstrap effects	3.341	0.001	0.070	0.292

*EWNB, engagement with natural beauty; DERS, difficulties in emotion regulation scale.*

## Discussion

The present evaluation considered the effects of taking part in *30 Days Wild* on happiness, health, conservation behaviors, nature connectedness, and EWNB to see if previous results were replicated. Then the analysis focussed on the relationships between happiness, EWNB, nature connectedness and emotional regulation.

### Does Taking Part in 30 Days Wild Have an Effect?

The results of the previous evaluation (Richardson et al., 2016a) were replicated with the analysis finding significant increases from pre-participation baseline to post-participation for nature connectedness, health, happiness, and conservation behaviors. There were also significant and sustained increases from pre-participation baseline to follow-up for the same measures. In a new finding, a significant and sustained increase in EWNB was found, making *30 Days Wild* the first intervention to increase EWNB (Diessner and Steiner, 2017). Although significant, the increase is modest, which can be explained by the scale measuring trait engagement with beauty (Diessner et al., 2008). As the first work to show an increase in EWNB, there is a need for further work to confirm such findings and consider the mechanism for the increase. For example, it is worth considering what the increase is in. The four questions in the EWNB scale are wide ranging, from noticing to physiological responses, emotion, spirituality, and aspects of nature connectedness. Therefore, it is possible that participating in *30 Days Wild* could increase sensitivity, or encourage participants’ to take greater notice of nature’s beauty, which may affect aspects of connectedness to nature, physiological and emotional responses. Or the campaign could help people notice their own physiological and emotional responses to nature, thus increasing engagement and appreciation of its beauty. Further quantitative and qualitative work could investigate these mechanisms.

### Does EWNB Mediate the Relationship Between Nature Connectedness and Well-Being?

The role of EWNB can be further considered through considering the relationship to well-being and nature connectedness. Further analysis showed that the increase in EWNB mediated the relationship between the increases in nature connectedness and happiness. The results provide support for the work of Zhang et al. (2014a) and Capaldi et al. (2017) showing that engagement with nature’s beauty is emerging as a key factor in the positive relationship between nature connectedness and well-being. Capaldi et al. (2017) propose that EWNB promotes nature connectedness to bring well-being. As noted above, the EWNB scale items include aspects of nature connectedness, noticing beauty, emotion, and spirituality. Indeed, there is a theoretical background that suggests nature’s beauty is a key part of the human relationship with nature. Aesthetics has been identified as a value of biophilia (Kellert, 1993), which mediates the relationship between compassion for nature and nature connectedness (Lumber et al., 2017) and beauty is a key theme when developing nature connectedness (Richardson and Hallam, 2013; Richardson et al., 2015). However, Zhang et al. (2014a) performed analysis that suggested the two were not a single construct. This is supported by the regression analysis which shows that EWNB, rather than nature connectedness, was a key predictor of the change in happiness.

Clearly, EWNB has a positive relationship with feelings such as happiness and the links between EWNB and emotion can be considered. Previous research (Richardson et al., 2016b; Chirico et al., 2017; Song et al., 2017) shows that engagement with nature and its beauty can be linked to affect regulation by considering the three-circle model of emotion (Gilbert, 2009). The three-circle model suggests that engaging with nature’s beauty can bring feelings of joy and calm, positive emotions that bring well-being through promoting emotional balance (Gilbert, 2009; Richardson et al., 2016b). Joy and calm can also be mapped onto the positive and relaxing reactions to nature noted by Ulrich (1983) and within psycho-physiological stress recovery theory (PSRT; Ulrich et al., 1991). This suggests that EWNB can bring well-being and restoration through emotional balance and reducing stress.

### What Is the Relationship Between Nature Connectedness, EWNB, Emotional Regulation, and Happiness?

Capaldi et al. (2017) suggest that EWNB affects well-being by promoting nature connectedness. Results from Gidlow et al. (2016) show that nature connectedness was not significantly correlated with restorative experience or cognitive function in green spaces, suggesting existing theories that explain the benefits of nature exposure (ART and PSRT) do not fully explain the benefits of nature connectedness. Therefore, there is a need to understand the mechanisms by which nature connectedness is linked to well-being. Given the affective relationship at the heart of nature connectedness and the need to note the role of nature in affect regulation (Korpela et al., 2018), supplementary data was collected to explore the links between nature connectedness, happiness, EWNB, and emotional regulation. The correlation analysis showed that those with difficulties in emotional regulation had a lower connection with nature and lower happiness. Interestingly, difficulty in emotional regulation was not associated with EWNB, supporting the distinction between EWNB and nature connectedness. However, a relationship might have been expected given the affect related questions in the EWNB scale and the responses to viewing nature’s beauty (Song et al., 2017). Mediation analysis indicated that emotional regulation mediated the relationship between nature connectedness and happiness, as EWNB mediated the relationship between nature connectedness and happiness in the earlier analysis. This is the first evidence linking affect regulation to the well-being benefits of nature connectedness. Consistent with the Polyvagal theory of Porges (2007) those people who have greater difficulties in affect regulation take longer to rebalance their emotions (Berna et al., 2014). Further, nature connectedness is known to be related to lower anxiety (Martyn and Brymer, 2016), a condition known to be associated with delayed physiological response following emotion elicitation (Berna et al., 2014). Finally, given EWNB and difficulty in emotional regulation were not related, but both have a role in mediating the relationship between nature connectedness and happiness, there is a suggestion that EWNB and emotional regulation interact with the relationship between nature connectedness and happiness through different mechanisms. Potentially, through affect regulation for nature connectedness and through cognitive mechanisms such as processing fluency (Reber et al., 2004) and being attuned (Kaplan, 1987) to nature’s beauty. However, these are likely to have complex and multi factorial underpinnings that require further research.

## Limitations and Conclusion

The evaluation of *30 Days Wild* is interesting as it is a public engagement campaign. However, despite good design practice, the evaluation does have limitations. These include the pre-post design (e.g., engagement EWNB was found to increase over time rather than in comparison to a control group) and the measures are intentionally short within the framework of a “Wildness Quiz.” There is also a high attrition rate, particularly from the baseline where the questions are an option from the sign-up process, rather a follow-up response by email. Therefore, the results may not fully reflect the outcomes of the majority of those taking part. The campaign is also self-directed and the activities of the 49,000 participants are not tracked, therefore adherence with the campaign cannot be formally measured, although given the attrition it is likely follow-up respondents are those who engaged with the campaign. The participants are also overwhelmingly female, which is an interesting finding in itself. Clearly, *30 Days Wild* appeals more to women than men and there is a need to explore the reasons for this and potential ways to engage men with nature for well-being. The large sample size did allow some significant differences between the genders to be identified, although proportionally these were relatively small, other than males scoring approximately ten percent higher on nature connectedness, which could well be a reflection of the higher age of male participants. These limitations mean that the results and conclusions should be treated with some caution, although the replication of core findings is positive. Finally, the scale and success of the campaign at a time when there are calls for large scale upstream nature based interventions for health warrants publication of the findings in order to inform the further research required.

To conclude, the replication of the improvements in nature connectedness, happiness, health, and conservation behaviors gives greater confidence in the success of *30 Days Wild* within a public health context. Further, the paper presents a significant and sustained increase in EWNB and previous research into the relationship between EWNB, nature connectedness and happiness is supported. The paper also presents new data on the links between nature connectedness, EWNB and affect regulation which gives some initial insight into the pathways to well-being. From an applied perspective, the relationships show that well-being in nature is not just about visits and exposure to nature. Rather, there is a need to engage in an affective relationship, to notice and become sensitive to nature’s beauty to access the wider benefits of nature connectedness and well-being.

## Data Availability

The raw data supporting the conclusions of this manuscript will be made available by the authors, without undue reservation, to any qualified researcher.

## Author Contributions

MR and KM contributed to the design of the evaluation. MR performed the statistical analysis and wrote the first draft of the manuscript. KM contributed to manuscript revision, read and approved the submitted version.

## Conflict of Interest Statement

The authors declare that the research was conducted in the absence of any commercial or financial relationships that could be construed as a potential conflict of interest.
